# Performance of Prenatal Ultrasound Screening for the Relative Positioning of Mesenteric Vessels

**DOI:** 10.1002/jum.16576

**Published:** 2024-09-11

**Authors:** Jean Michel Faure, Anne Larroque‐Devigne, Dominique Forgues, Eve Mousty, Alain Couture, Nicolas Kalfa, Olivier Prodhomme, Florent Fuchs

**Affiliations:** ^1^ Department of Obstetrics and Gynecology University Hospital Center Montpellier France; ^2^ Department of Abdominal and Urologic Surgery CHU Montpellier, Hôpital Lapeyronie Montpellier France; ^3^ Department of Obstetrics and Gynecology University Hospital Center Nîmes France; ^4^ Department of Pediatric Imaging CHU Montpellier Montpellier France; ^5^ Inserm, CESP Center for research in Epidemiology and Population Health U1018, Reproduction and Child Development Villejuif France; ^6^ Desbret Institute of Epidemiology and Public Health University of Montpellier Montpellier France

**Keywords:** intestinal malrotation, superior mesenteric artery, superior mesenteric vein, superior mesenteric vessels positions, ultrasound prenatal diagnosis

## Abstract

**Objectives:**

Abnormal relative positioning of the superior mesenteric artery (SMA) and vein (SMV) can lead to intestinal malrotation that predisposes to midgut volvulus. The aim of this study was to assess the prenatal ultrasound ability to visualize the relative position of SMA and SMV in normal pregnancies.

**Methods:**

Prospective cohort study performed in Montpellier University Hospital Centre, including 80 fetuses during routine 3rd trimester ultrasound scan. For each fetus included, the relative position of the vessels on an axial image was defined as SMV on the right, forward, or on the left of SMA. Doppler imaging was additionally used if necessary. Data were compared to the neonatal abdominal scans performed by pediatric radiologist.

**Results:**

The superior mesenteric vessels were identified in 79 fetuses. Prenatal findings showed a usual relative position of the vessels, that is, the vein on the right of the artery, in 96.2%. In 2 cases, the vein was strictly in front of the artery, and in 1 case, the vein was on the left side of the artery. Seventy‐four neonates were examined and comparison with prenatal finding showed a perfect agreement (Kappa coefficient of 100%). An intestinal malrotation was postnatally diagnosed corresponding to the case where vein was on the left side of the artery.

**Conclusion:**

This study showed that the relative position of the SMA and SMV could be assessed using ultrasound prenatal examination with a perfect agreement with postnatal findings. In case of abnormal vessels positioning more examinations should be promote including prenatal MRI and postnatal conventional radiologic examinations to confirm intestinal malrotation.

**Abbreviations**
SMA: superior mesenteric artery
SMV: superior mesenteric vein
US: ultrasound

Intestinal malrotation corresponds to an abnormal rotation of the intestine which occurs during the embryonic period. It involves the midbowel, which is defined by the midgut part that goes from the distal duodenum to the midtransverse colon. Midgut malrotation is known as a dangerous situation, because of the risk of midgut volvulus, where the first jejunal loop and terminal ileum are close with very short mesenteric root. Its actual incidence is not really known and goes from 1/200 to 1/6000.^1^ Midgut volvulus is commonly diagnosed after birth when the milk inflow, increasing the bowel weight, imbalances the mesentery resulting in its torsion. On the opposite, fetal midgut volvulus secondary to intestinal malrotation is a very rare condition.^2^, ^3^ Midgut volvulus is a great surgical emergency of the neonate or childhood as it can lead to intestinal lumen obstruction resulting in venous and arterial ischemia.^4^, ^5^, ^6^

When a newborn is suspected to have an occlusive midgut syndrome, the diagnosis of malrotation is suggested by ultrasound (US) exploration of the relative positioning of the superior mesenteric vessels due to its link with intestinal rotation.^7^, ^8^, ^9^, ^10^, ^11^, ^12^ The superior mesenteric vessels are explored by an axial transverse abdominal view, and are followed downward from their origin. The US diagnosis of malrotation is based on an abnormal relative positioning of superior mesenteric artery (SMA) and vein (SMV): the SMV lying at its left side. It is completed with radiological upper gastrointestinal series and barium enema to assess Treitz angle and caecum positioning in most countries.^13^, ^14^, ^15^, ^16^

Although cases of antenatal midgut volvulus secondary to malrotation syndrome had been reported after delivery,^3^, ^17^, ^18^ no antenatal screening of malrotation has ever been reported. However, screening prenatally for bowel malrotation before the occurrence of a complication could help pediatricians for medical survey of the newborn at risk.^19^

The aim of this study was to evaluate the ability of prenatal US examination to identify the relative position of the SMA and SMV in comparison with the gold standard being postnatal US examination of the newborn.

## Materials and Methods

From September 2022 to February 2023, all pregnant women aged over 18 years, with a singleton pregnancy of more than 31 weeks of gestation, referred to our University Center Hospital in Montpellier (France) for a routine 3rd trimester US scan were asked to participate in this cohort study after informed consent. Noninclusion criteria were the existence of a malformative syndrome on US, heterotaxia with asplenia or polysplenia, diaphragmatic hernia, omphalocele, gastroschisis, gastrointestinal pathology, abdominal tumor, bladder exstrophy, megacystis, or chromosomal abnormality, or a patient with a body mass index >30 kg/m^2^ at the beginning of the pregnancy. Pregnancy dating was based on crown‐rump length measurement during the 1st trimester US.

US examinations were performed by a single, trained and experienced sonographer and were obtained with a General Electric Voluson E10 (GE Medical Systems®, Zipf, Austria) machine, equipped with an abdominal multiple frequency RM6C probe.

**Figure 1:**
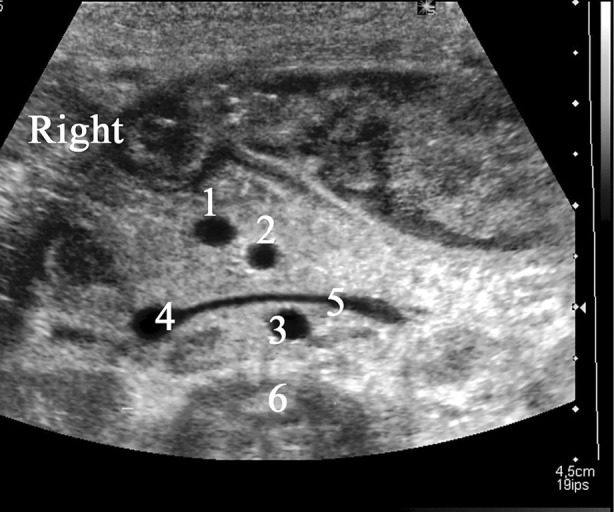
Pediatric abdominal ultrasound showing the gold standard section to analyze superior mesenteric vessels location. The superior mesenteric vein (SMV) (1) is normally on the right side of the superior mesenteric artery (SMA) (2). The two mesenteric vessels are visualized in a strict cross‐sectional plane. Between the aorta (3) and the SMA (2) the left renal vein (5) draining into the inferior vena cava (4) is visualized. Spine (6).

**Figure 2:**
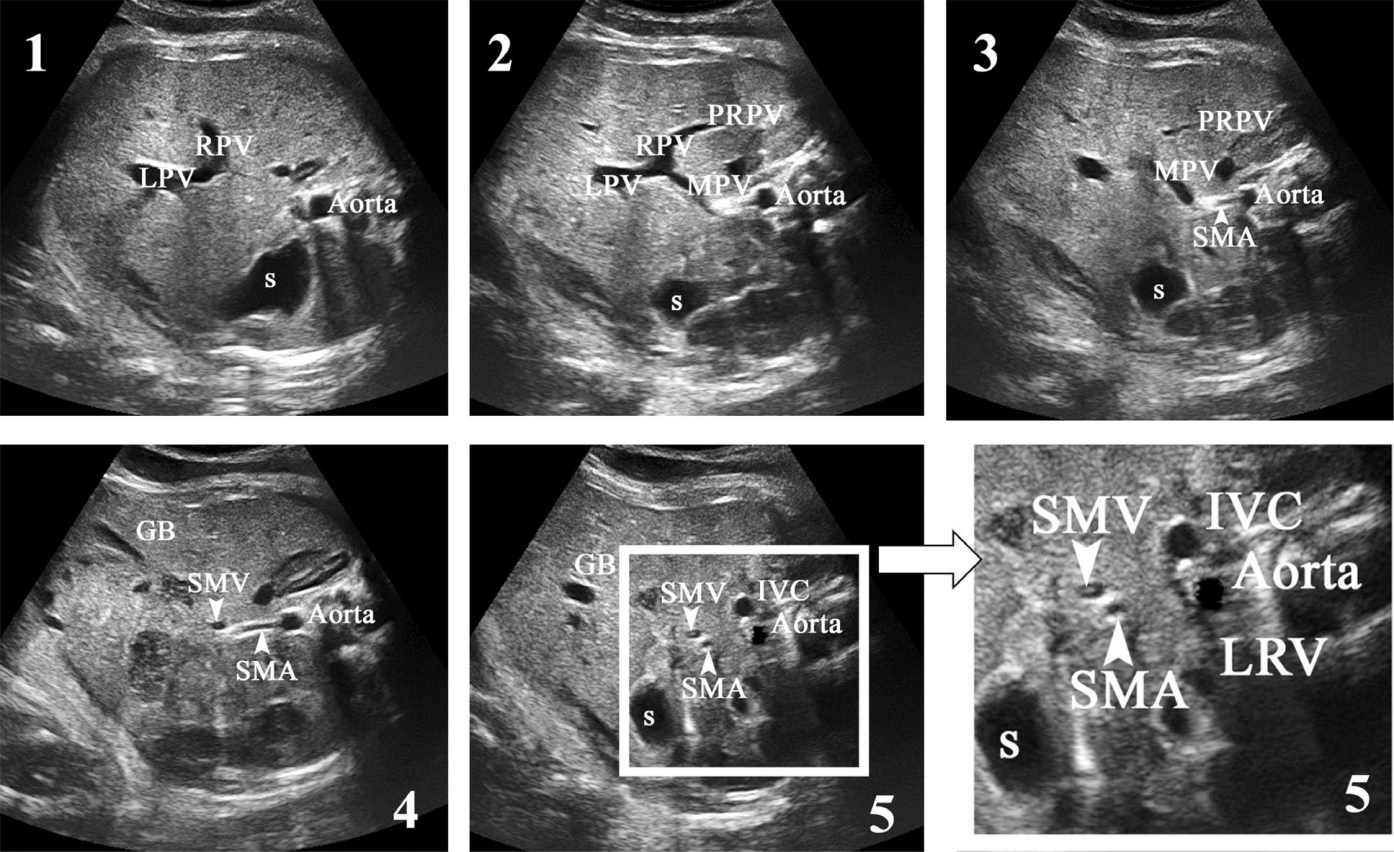
Prenatal ultrasound imaging of the fetal abdomen (transversal view). The left side of the fetus corresponds to the bottom of the image. The portal sinus, corresponding to the junction of the left portal vein (LPV) and the right portal vein (RPV), is visible on the cross‐section of the abdominal circumference (1). The main portal vein (MPV) (2 and 3) arise from the portal sinus with an oblique left and inferior direction and continues with the superior mesenteric vein (SMV) (4). The superior mesenteric artery (SMA) has a peripheral echogenic halo. It originates from the anterior part of the aorta and goes anteriorly and downwards (4). On the reference ultrasound plane (5), the two mesenteric vessels travel in close proximity to each other and are visualized in a strict cross‐sectional plane. Ideally, between the aorta and the SMA the left renal vein (LRV) draining into the inferior vena cava (IVC) is visualized. Posterior branch of right portal vein (PRPV). S, stomach; GB, gallbladder.

**Figure 3:**
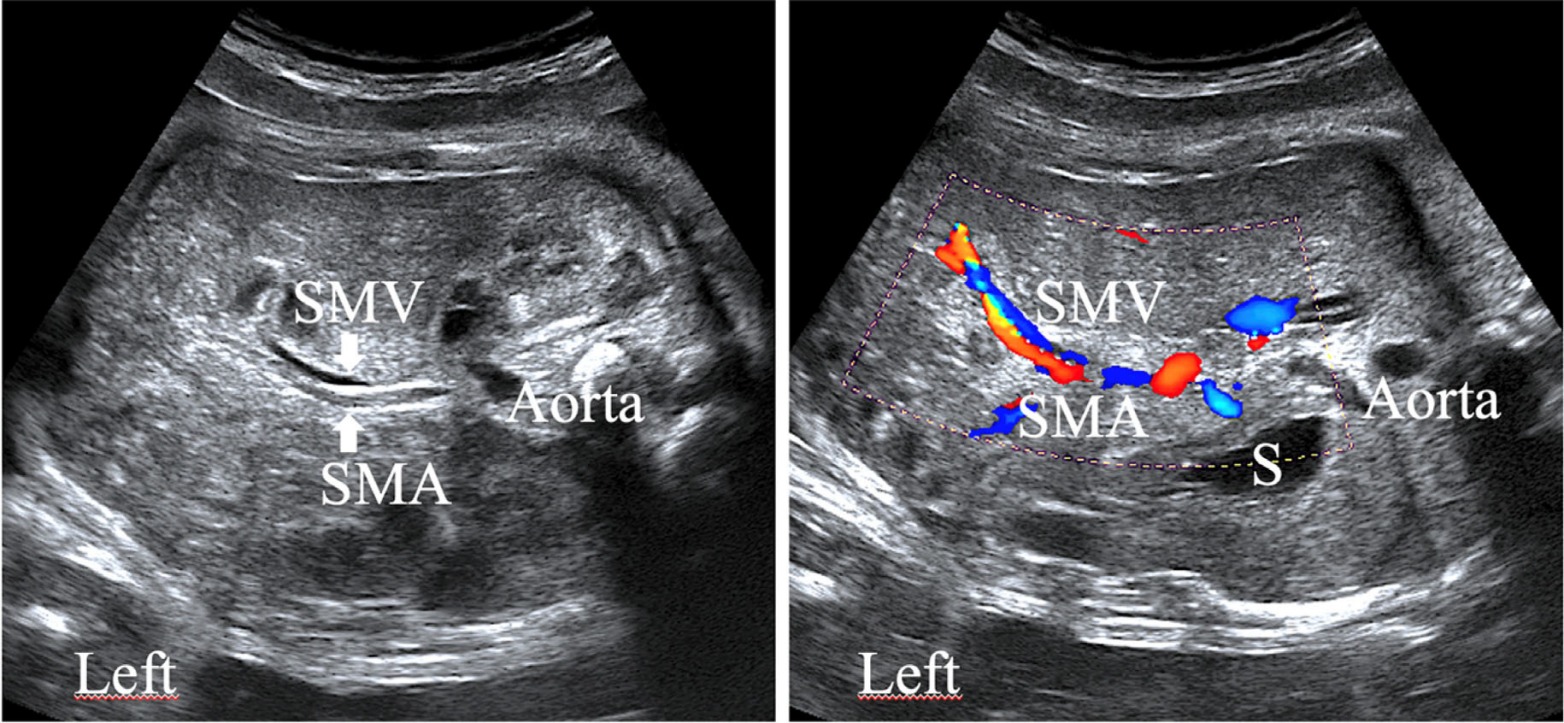
Prenatal ultrasound imaging of the fetal abdomen (transversal view). The branches of the mesenteric vessels are usually located at the right part of the fetal abdomen. Color Doppler flow could be used for better vessels detection. The SMV appears in blue and SMA in red (identically to inferior vein cava and aorta, with a same orientation of the probe). S, stomach.

After determining fetal positioning and abdominal situs, the US plane defining the abdominal circumference was obtained. To obtain the anatomical transversal plane to analyze the relative positioning of the superior mesenteric vessels, we applied the same methodology as the one previously described in neonates (Figure 1). Starting from the abdominal circumference plane, the transducer was slightly translated downward toward fetal feet. The different anatomical landmarks corresponding to the quality criteria of the image are detailed in Figure 2: from backward to forward we can describe the spine, the abdominal aorta on its left, the inferior vena cava on its right, the left renal vein, SMA/SMV left sided to the spine, gallbladder, and umbilical vein on above with slow deviation on the right. Vessels exploration begins with B‐mode. The vessels are anechoic. The SMA has a peripheral halo well seen in the 3rd trimester, it arises from the anterior aortic wall and runs downward. Between abdominal aorta and the SMA, the left renal vein running into inferior vena cava can be seen. The SMV, that present a greater diameter than the SMA, is running downward from the portal confluent typically right sided to SMA. Lower in the abdomen, branches of mesenteric vessels can also be seen, normally running to the right part of the fetal abdomen (Figure 3). Color Doppler flow and pulse wave Doppler were used for better vessels detection and identification. For each fetus included, the fetal positioning, the placental position, and the time taken to perform the fetal US examination were recorded.

After delivery, all newborns were referred to a senior pediatric radiologist for US examination performed between J0 to J2 of life, to determine the relative position of the superior mesenteric vessels. The senior pediatric radiologist was blind from prenatal results.

The data are presented in n (%) or mean ± SD (range). The postnatal findings were compared with the prenatal US data for each fetus studied. The comparison was made using postnatal pediatric examination performed by transabdominal approach constituting the gold standard. If the vessels origin was hidden due to bowel distension, color flow and pulse wave Doppler were used. The statistical analysis was performed using SAS software (version 9.1). Descriptive statistics were performed using the usual classical methods and then rendered, for qualitative variables, as a percentage with 95% confidence interval. The statistical tests were carried out with a bilateral approach and a risk of error of first alpha species set at 5%. The normality test used was the Shapiro–Wilk test. Kappa coefficient was used to compare pre‐ and postnatal findings and evaluate the agreement. A test was considered significant when its significance level *P* was below the alpha significance level (*P* < alpha or *P* < .05).

This study received ethics approval by the Montpellier University Hospital Institutional Review Board under the number: IRB‐MTP_2021_11_202100980.

## Results

From September 2022 to February 2023, 86 patients fulfilled inclusion criteria and 80 fetuses (93%) were finally included during the 3rd trimester US examination after informed consent. Among those, one case was excluded because of poor maternal conditions and fetal anterior position of the back. In the study population, the mean maternal age was 31.4 ± 5.2 years and 52% of patients were primiparous. The mean body mass index at US examination was 23.94 ± 4.1 kg/m^2^. The mean gestational age at US examination was 31.6 weeks (30–32.9 weeks).

All fetuses were in cephalic position. The position of the back was on the left in 44%, right in 50%, and backward in 6% of cases. Situs determination was normal for all 79 fetuses studied. The placenta was posterior in 56% of cases, and anterior in 44%. The median duration of the entire US scan was 19 ± 5.7 minutes (15–27). The mean time to the mesenteric vessels detection and exploration was under 1 minute in 74 cases and over 1 minute in 5 cases.

**Figure 4:**
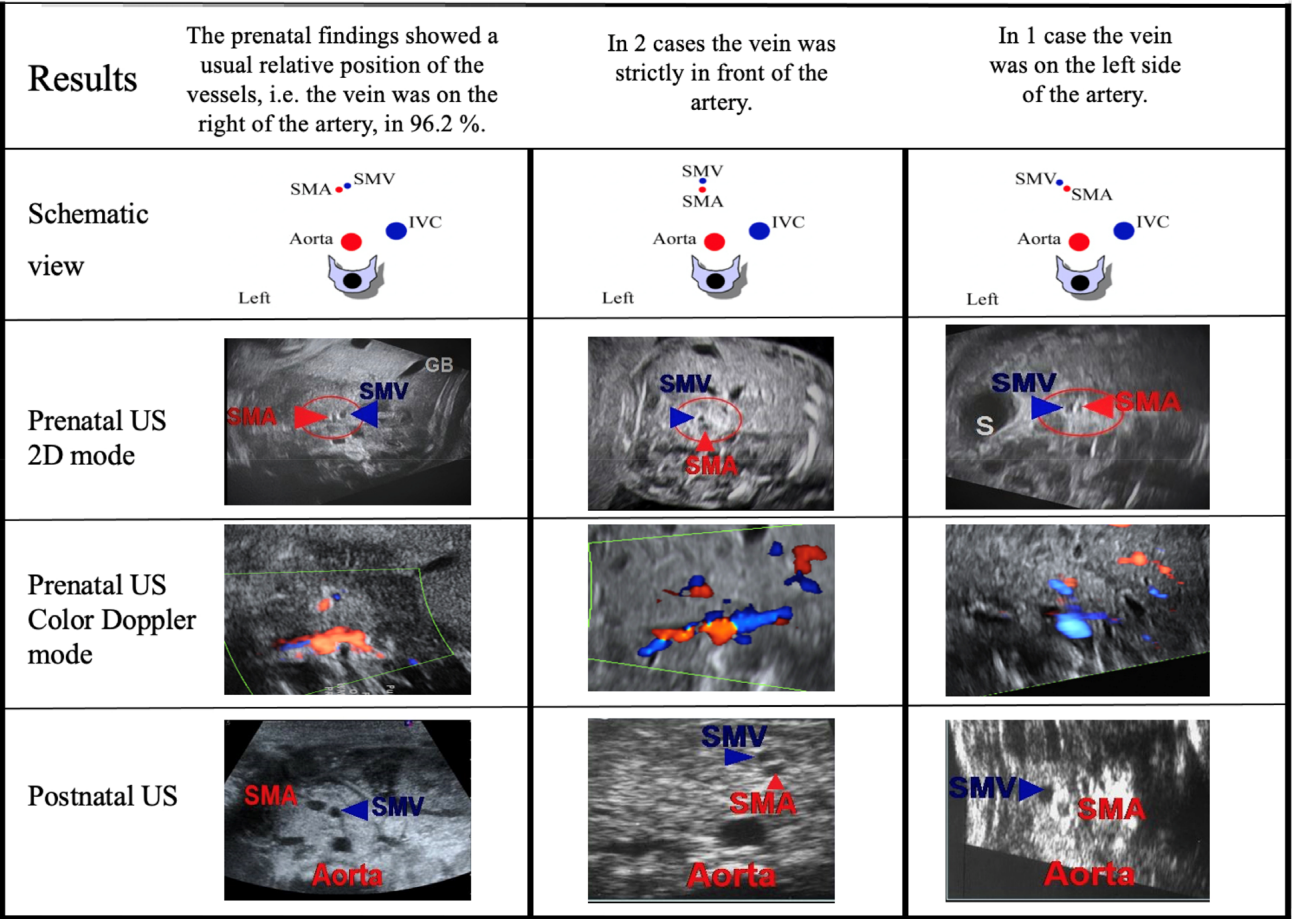
Pre‐ and postnatal ultrasound findings of mesenteric vessels positioning.

The SMV was recognized in all 79 fetuses; in 76 (96.25%) cases the SMV was right sided to the SMA, in 2 (2.5%) cases the vein was forward of the artery and in 1 (1.25%) case the SMV was left sided to the artery (Figure 4). For 75 fetus the position of the SMV was detected only using B‐mode. In 4 cases, due to poor visualization on B‐mode, a color‐flow and pulse wave Doppler were used to detect the two vessels (Table 1).

**Table 1:** Ultrasound Relative Position of Mesenteric Vessels Between Prenatal and Postnatal

	Relative Position of Mesenteric Vessels	n (%)	95% Confidence interval
Prenatal ultrasound	SMV on the right of SMA	76 (96.20)	88.55–99.01
	SMV in front of SMA	2 (2.53)	0.44–9.69
	SMV on the left of SMA	1 (1.27)	0.07–7.82
	Total	79	
Postnatal ultrasound	SMV on the right of SMA	71 (95.95)	87.82–98.95
	SMV in front of SMA	2 (2.70)	0.47–10.3
	SMV on the left of SMA	1 (1.35)	0.07–8.32
	Total	74	

From all 79 fetuses included only 74 (94%) newborns were followed after delivery and 5 missed their exploration. For these five cases, the prenatal US screening has showed a right‐sided position of the SMV to the SMA. Postnatal imaging revealed 71 (96%) cases with the vein positioned right sided to the artery, 2 cases above and at the midline (3%) and in 1 case the vein was on the left arterial side (1%). The vascular landmarks seen in prenatal US scans were totally similar to those identified in the newborns (Table 2) leading to a Kappa coefficient of 100%. This corresponded to a perfect agreement according to Landis and Koch.^20^

**Table 2:** Anatomical Concordance Between Pre‐ and Postnatal Ultrasound

Postnatal	SMV on the Right of SMA	SMV in Front of SMA	SMV on the Left of SMA
Prenatal			
SMV on the right of SMA	71	–	–
SMV in front of SMA	–	2	–
SMV on the left of SMA	–	–	1

Considering the high risk of volvulus in case of prenatal screening with the SMV on the left side of the SMA, it was decided after counseling of the Multidisciplinary Prenatal Diagnosis Center to perform further prenatal examinations after information and agreement of the patient. A fetal abdominal MRI was performed after 32 weeks. T2‐w sequences showed the liquid content in the jejunal loops located in the right part of the abdomen at the opposite side of the stomach (in the left side). T1‐w sequences documented a medial subhepatic caecum. MRI offered the ability to suspect in this case, an incomplete common mesentery, abnormality at high risk of neonatal volvulus. A surgical prenatal counseling and neonatal US examination completed by upper gastrointestinal series and barium enema were performed. Arguments were made for a malrotation because the duodenum–jejunal junction was on the right of the spine. Short mesenteric root was suspected because the caecum was on the midline subhepatic position. This was the first case of uncomplicated midgut malrotation secondary to common incomplete mesentery type prenatally diagnosed.

## Discussion

This is the first prenatal sonographic study that try to screen midgut malrotation using the assessment of the relative position of the superior mesenteric vessels. These vessels were completely identified in this prenatal study showing that the relative position of the SMA and SMV could be assessed using US prenatal examination with a perfect postnatal agreement. In most of the cases, the B‐mode was enough to confirm the vessels anatomy and their relationship. In only few cases (5%), visualization of the vessels was uncertain and additional use of color flow and pulse wave Doppler were necessary.

**Figure 5:**
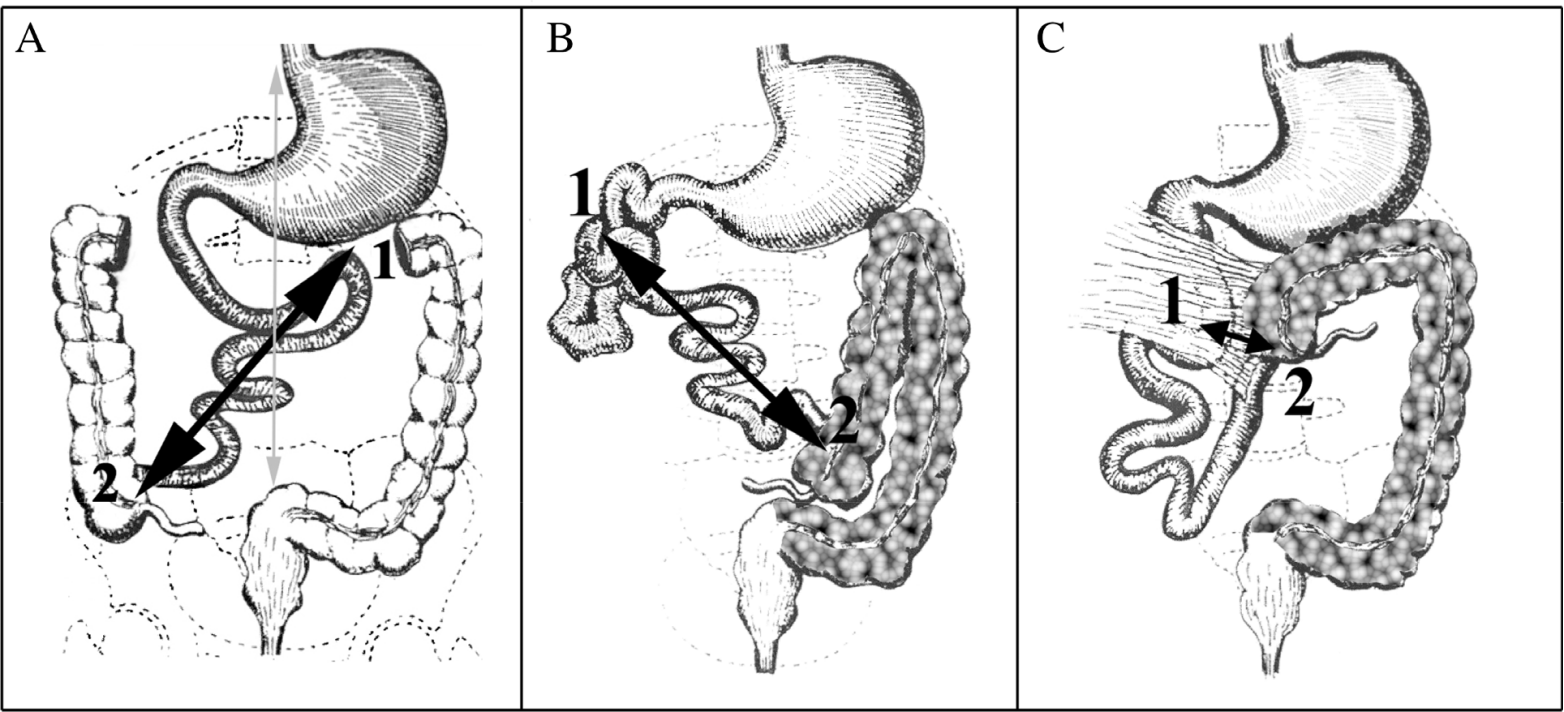
**A**, Normal bowel rotation. The Treitz angle (1) is on the left side of the spine and the ileocaecal junction (2) in the right flank. The mesenteric root is broad. **B**, Complete common mesentery type. The duodenojejunal junction (1) and the jejunum are to the right of the spine. The ileocaecal junction (2) lies on the left inferior position. The mesenteric root is then large but inverted from upper right to lower left. **C**, Incomplete common mesentery type. The duodenojejunal junction (1) and the jejunum are to the right of the spine. The ileocecal junction (2) is joined in the subhepatic region in a median position by the duodenocolic peritoneal Ladd's bands. The mesenteric root is narrow.

Embryological process leading to normal intestinal rotation is complex. It is based on a rotation from outside to inside the fetal abdomen in a counterclockwise of 270° around the SMA axis and during three different stages. During the first stage (between 5 and 10 weeks), there is a physiological herniation of the bowel into the umbilical cord. The proximal midgut loop corresponding to duodenojejunal loop, located upper and in the midline to the SMA, rotates at a 90° counterclockwise to become horizontal. In the next 2 weeks, it moves once again at a 90° counterclockwise (totally at 180°) and reaches the midline at the right site to the SMA. In the same time, the distal loop corresponding to caecocolic loop, located beneath the SMA, rotates at a 90° counterclockwise and ends at the left side of the last one. Both loops maintain this position until the bowel returns to the abdominal cavity. The second stage occurs at 10 weeks. The bowel returns into the abdominal cavity. During this period the duodenojejunal loop rotates at a 90° counterclockwise and ends at the left side to the SMA. The caecocolic loop subsequently turns more at 180° when it returns back to the abdominal cavity, and comes right sided to SMA. Finally, during the third stage (between 11 and 12 weeks), descending of the caecocolic loop and fixation of the mesenteries occur. At the end of the normal physiological rotation, the midgut is fixed to the posterior abdominal cavity: the duodenum is fixed by Treitz fascia, the duodenojejunal loop is fixed in the upper side of mesentery roof, left sided to the SMA, and finally, the caecocolic loop is fixed diametrically opposed to the upper segment, into the lower right abdominal quadrant by Todt's fascia (Figure 5A).

During those three stages, the midgut grows up and lengthens along the SMA. With the normal rotation, a broad pedicle is achieved at the base of SMA and protects against midgut volvulus. SMA vascularizes small bowels from the third duodenum to the left colic flexure. It arises from the aorta just below the origin of the coeliac trunk and goes down to the left renal vein behind the pancreas. Below, it goes on the right and crosses the third duodenal and in the mesenteric root, stays on the left side of the SMV. SMV begins above terminal ileum and goes up on mesenteric root. The SMV is normally on the right of the SMA. The SMV ends behind the pancreatic isthmus, and unites with the spleno‐mesaraic trunk to create the portal vein.

In case of an abnormal midgut rotation, two types of situation can be seen, with for both, the SMV left sided to the SMA. The complete common mesentery type corresponds to a stop after the first rotation at a 90° (Figure 5B). As a result, the duodenum and duodenojejunal loop lie down at the right of SMA, the caecocolic loop lies at its left side. The mesenteric root is inverted from upper right for the duodenojejunal loop to lower left for the caecocolic loop. The root is horizontal or oblique with broad base. This situation is not symptomatic and has a very low risk of volvulus. On the opposite, the incomplete common mesentery type corresponds to a stop after rotation at a 180° of the upper part of the midgut and rotation of the 90° up to 180° of its lower part (Figure 5C). The duodenojejunal loop lies down at the right of the SMA at midline, the caecocolic loop lies usually on the left of SMA and beneath the liver. Consequently, the mesenteric root is tethered by the narrow stalk, which can twist around itself and prone to high risk of volvulus. The Ladd's bands can extend between the caecocolic loop and the upper right abdominal quadrant, across the duodenum with possible obstruction of the last one. Usually symptomatic, this malrotation appears in the new‐born and during the first year as an upper obstructive syndrome, either of duodenal compression by the Ladd's bands, or, most dramatically with volvulus. The volvulus in the incomplete type malrotation is prone to intestinal ischemia (venous or arterial), leading to bowel necrosis.

In our study, we aim to diagnose unusual positioning of mesenteric vessels as it is associated with increased risk of abnormal midgut rotation. In 100% cases the position of the mesenteric vessels described antenatally was similar to that described postnatally (Figure 4).

**Figure 6:**
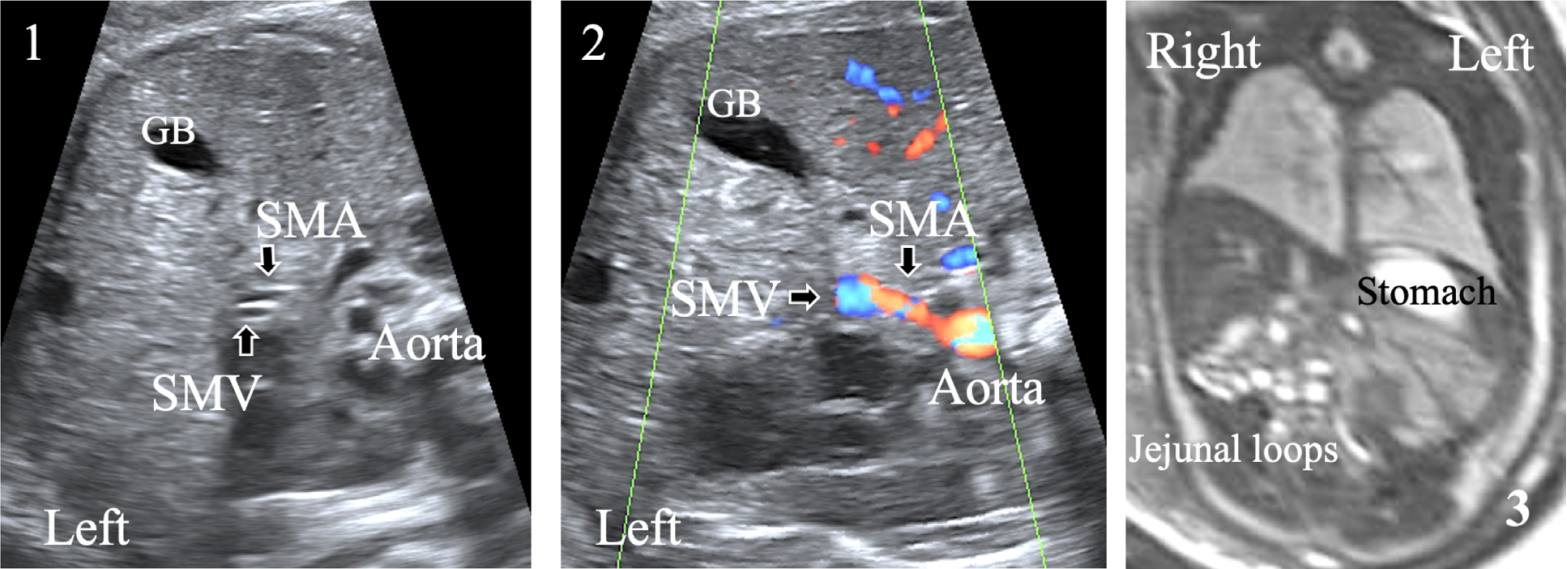
Midgut malrotation. Prenatal ultrasound imaging of the fetal abdomen (transversal view). Abnormal antenatal position (1): the superior mesenteric vein (SMV) is on the left side of the artery (SMA). Color Doppler flow is used for better vessels detection. The SMV appears in blue and SMA in red (2). GB, gallbladder. Prenatal fetal MRI (3), T2‐w sequence. It confirms the abnormal position of the jejunal loops (liquid content, hypersignal) located in the right flank, below the liver. The stomach is normally located is on the left.

- The SMV is right‐sided by the SMA (n = 71). This position is the most frequently seen in normal mesenteric rotation. This position is not considered at risk of volvulus secondary to malrotation.
- The SMV is located in front of, on the midline or partially on the left side of the SMA (n = 2). In these two cases the prenatal US examination shows an abnormal relationship between the mesenteric vessels. The same data were confirmed by postnatal US examination. The SMV appeared above and midline to the SMA into the upper vascular pedicle, but at the lower levels, there was completely normal relationship with SMV lying down on the right of the SMA. This topography is not compatible with real malrotation syndrome and more frequently is seen in nonrotation or complete common mesenteric syndrome. That is why, we emphasize the importance of complete US follow‐up of the superior mesenteric vessels from the beginning of the SMA at the anterior part of the aorta to its distal terminations which are normally located on the right of the fetal abdomen (i.e., normal place of intestinal ileal loops).
- The SMV lies at the left side of the SMA (n = 1). In this case, the prenatal US performed at 33 weeks showed the typical abnormality of the mesenteric vessels's relationship: the SMV lies down to the inner left arterial side, very suggestive to midgut malrotation syndrome. After delivery, abdominal US, upper gastrointestinal series and barium enema, confirmed the diagnosis of incomplete malrotation with high risk of volvulus because of narrow mesentery stalk (Figure 6).

**Figure 7:**
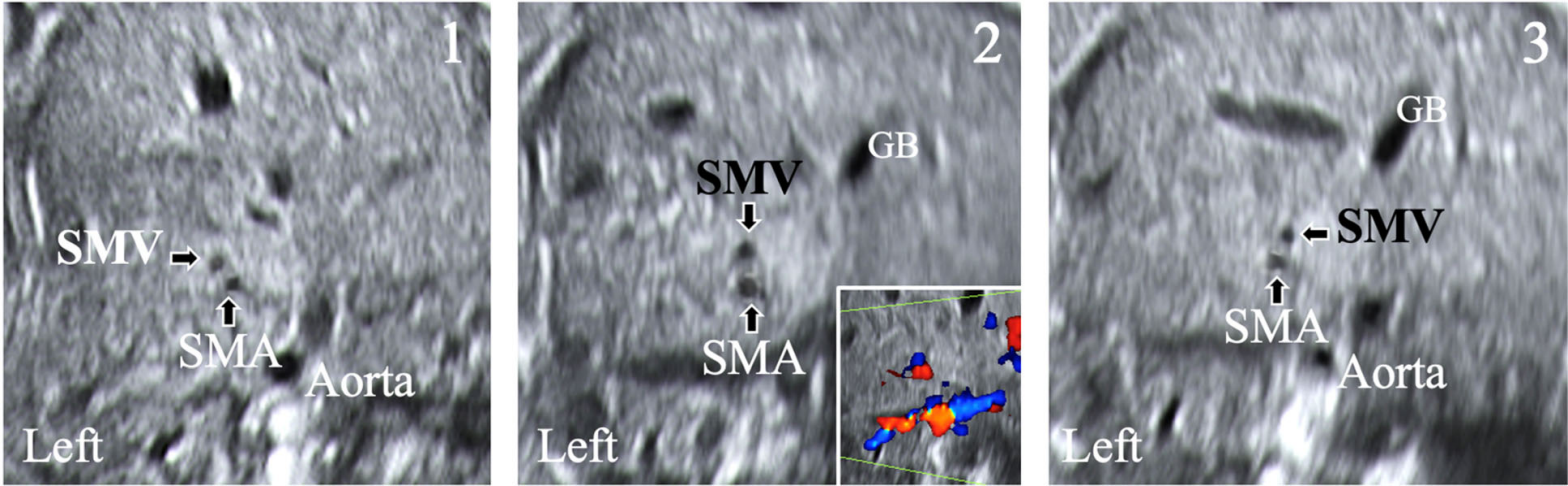
Prenatal ultrasound imaging of the fetal abdomen (transversal view). Prenatal ultrasound of “crazy” vein. The SMV appears initially on the left (1), then in front of and medial to the artery at the level of the reference section (2). Below the vein appeared in normal position, to the right of the artery (3). GB, gallbladder.

**Figure 8:**
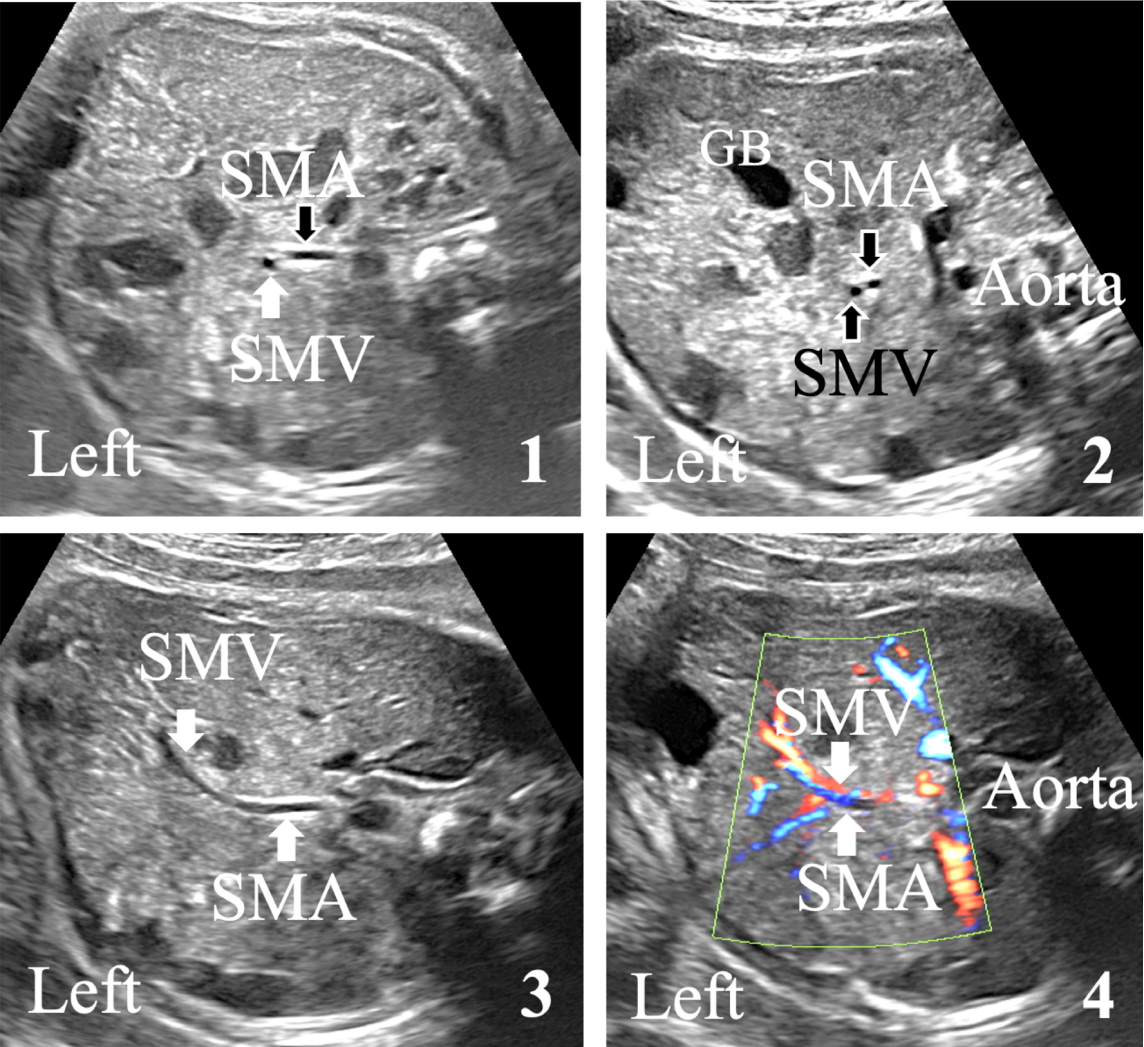
Prenatal ultrasound imaging of the fetal abdomen (transversal view). Prenatal ultrasound of false positive case. The SMV appears initially on the left (1), then in front of the artery (2). The branches of mesenteric vessels have a normal pathway to the right part of the fetal abdomen (3). Color Doppler mode (4) offers correction of technical errors. GB, gallbladder.

During the learning period of the method, some difficulties were met to locate precisely the relative position of the vein to the artery. In two cases the vessels initially appeared to be wrongly positioned: one case of median vein and another case with an “S” pathway; the vein initially positioned on the left side then finally on the right side of the artery (Figure 7). This was due to errors in obtaining the correct anatomical section plane. In the first case, the section plane was too high, and the vein visualized was not VMS but the spleno‐mesaraic trunk. In the second case, the section plane studied was too oblique and the interpretation of the relative position of the vessels was not made in relation to the recommended anteroposterior median axis. Nevertheless, after use of the color Doppler mode and after correction of technical errors in order to be in the correct ultrasonographic study plane, the vein was finally found to the right in both cases in the prenatal study. Postnatally, the vein was confirmed to be on the right side in both cases. This underlines the rigor required to obtain the US reference section plane, especially the need of visualization of the left renal vein on this studied plane. In another one case, prenatal US described a vein located in front of and medial to the artery at the level of the reference section (Figure 8). However, below this section, the vein appeared in normal position, to the right of the artery. Postnatal ultrasonography confirmed that the vein was indeed forward and medial to the artery at the level of the origin of the mesenteric vessels, and that further down the vein had a normal path to the right, as far as the division branches. This situation is known in newborns, and reflects the value of following the mesenteric vessels from their origin to their divisional branches in case of a malposition.^21^

The US prenatal study of the relative position of superior mesenteric vessels for an intestinal malrotation screening can be part of the morphological assessment of some fetal malformations. Intestinal malrotation may be the cause of duodenal stenosis secondary to the existence of Ladd's bands and must be searched in this condition. The midgut malrotation, even in its less severe form (complete common mesentery), is usually associated with other anomalies of intestinal integration such as omphalocele, laparoschisis et diaphragmatic hernia in whom the volvulus is fairly often, due probably to the shortened of the mesenteric root and intraperitoneal adhesions.^22^, ^23^, ^24^ The study of the superior mesenteric vessels must be also assessed when an heterotaxia is diagnosed or suspected in order to complete the fetal screening.^25^, ^26^, ^27^, ^28^ Malrotation of the bowel is also associated with a number of syndromes and other anomalies prone to volvulus, such as Prune–Belly syndrome and cloacal or bladder exstrophy.^29^ A biliary atresia syndrome should be suspected if the malrotation is associated with nonvisualization of the gallbladder.^30^ In case of an over distention of the bladder associated to an abnormal position of superior mesenteric vessels, a megacystis–microcolon–intestinal hypoperistalsis syndrome should be suspected.^31^ Recently the impact of SMA doppler has been assessed in comparison between hyperechoic and normal bowel.^32^

Considering the high concordance of the anatomic landmarks, we suppose that the prenatal US determination of the relative position of the superior mesenteric vessels could help us for prenatal screening of midgut malrotation and a prospective study could be done to confirm these data, especially during the second trimester US examination.

## Conclusion

This study showed that the relative position of the SMA and SMV could be defined using 30–34 weeks US prenatal examination with an excellent postnatal concordance. In case of an abnormality, more examinations should be done, including prenatal MRI and postnatal conventional radiologic examinations, to confirm intestinal malrotation. An earlier diagnosis might prevent complications including bowel ischemia, necrosis, and death. Screening for abnormal relationship of upper mesenteric vessels could also be used in the diagnosis of some syndromes and multiple fetal malformations.

## Data Availability

Data sharing not applicable to this article as no datasets were generated or analysed during the current study.

## References

[1] Kapfer SA , Rappold JF . Intestinal malrotation not just the pediatric surgeon's problem. J Am Coll Surg 2004; 199:628–635. 15454150 10.1016/j.jamcollsurg.2004.04.024

[2] Molvarec A , Babszinski A , Kovacs K , Toth F , Szalay J . Intrauterine intestinal obstruction due to fetal midgut volvulus: a report of two cases. Fetal Diagn Ther 2006; 22:38–40. 17003554 10.1159/000095841

[3] Yoo SJ , Park KW , Cho SY , Sim JS , Hban KS . Definitive diagnosis of intestinal volvulus in utero. Ultrasound Obstet Gynecol 1999; 13:200–203. 10204213 10.1046/j.1469-0705.1999.13030200.x

[4] Berdon WE , Baker DH , Bull S , Santulli TV . Midgut malrotation and volvulus. Which film are most helpful? Radiology 1970; 96:375–383. 5431424 10.1148/96.2.375

[5] Strouse PJ . Disorders of intestinal rotation and fixation (“malrotation”). Pediatr Radiol 2004; 34:837–851. 15378215 10.1007/s00247-004-1279-4

[6] Torres AM , Ziegler MM . Malrotation of the intestine. World J Surg 1993; 17:326–331. 8337878 10.1007/BF01658699

[7] Dufour D , Delaet MH , Dassonville M , Cadranel S , Perlmutter N . Midgut malrotation, the reliability of sonographis diagnosis. Pediatr Radiol 1992; 22:21–23. 1594305 10.1007/BF02011604

[8] Orzech N , Navarro OM , Langer JC . Is ultrasonography a good screening test for intestinal malrotation ? J Pediatr Surg 2006; 41:1005–1009. 16677901 10.1016/j.jpedsurg.2005.12.070

[9] Zhou L , Li S , Wang W , et al. Usefulness of sonography in evaluating children suspected of malrotation. Comparison with an upper gastrointestinal contrast study. J Ultrasound Med 2015; 34:1825–1832. 26362146 10.7863/ultra.14.10017

[10] Weinberger E , Winters WD , Liddell RM , Rosenbaum DM , Krauter D . Sonographic diagnosis of intestinal malrotation in infants: importance of the relative positions of the superior mesenteric vein and artery. AJR Am J Roentgenol 1992; 159:825–828. 1529850 10.2214/ajr.159.4.1529850

[11] Zerin JM , DiPietro MA . Superior mesenteric vascular anatomy at US in patients with surgically proved malrotation of the midgut. Radiology 1992; 183:693–694. 1584922 10.1148/radiology.183.3.1584922

[12] Esposito F , Vitale V , Noviello D , et al. Ultrasonographic diagnosis of midgut volvulus with malrotation in children. J Pediatr Gastroenterol Nutr 2014; 59:786–788. 25023580 10.1097/MPG.0000000000000505

[13] Sizemore AW , Rabbani KZ , Ladd A , Applegate KE . Diagnostic performance of the upper gastrointestinal series in the evaluation of children with clinically suspected malrotation. Pediatr Radiol 2008; 38:518–528. 18265969 10.1007/s00247-008-0762-8

[14] Sodhi KS , Khandelwal N . Upper gastrointestinal studies in malrotation. Pediatr Radiol 2008; 38:1034. 18607584 10.1007/s00247-008-0926-6

[15] Applegate KE , Anderson JM , Klatte EC . Intestinal malrotation in children: a problem‐solving approach to the upper gastrointestinal series. Radiogr Rev Publ Radiol Soc N Am Inc 2006; 26:1485–1500. 10.1148/rg.265055167 16973777

[16] Nguyen HN , Navarro OM , Bloom DA , et al. Ultrasound for midgut malrotation and midgut volvulus: *AJR* expert panel narrative review. AJR Am J Roentgenol 2022; 218:931–939. 35107311 10.2214/AJR.21.27242

[17] Crisera CA , Ginsburg HB , Gittes GK . Fetal midgut volvulus presenting at term. J Pediatr Surg 1999; 34:1280–1281. 10466613 10.1016/s0022-3468(99)90169-0

[18] Sabharwal G , Strouse PJ , Islam S , Zoubi N . Congenital short gut syndrome. Pediatr Radiol 2004; 34:424–427. 14676985 10.1007/s00247-003-1087-2

[19] Graziano K , Islam S , Dasgupta R , et al. Asymptomatic malrotation: diagnosis and surgical management: an American pediatric surgical association outcome and evidence‐based practice committee systematic review. J Pediatr Surg 2015; 50:1783–1790. 26205079 10.1016/j.jpedsurg.2015.06.019

[20] Landis JR , Koch GG . The measurement of observer agreement for categorical data. Biometrics 1977; 33:159–174. 843571

[21] Zerin JM , DiPietro MA . Mesenteric vascular anatomy at CT: normal and abnormal anatomy appearances. Radiology 1991; 179:739–742. 2027985 10.1148/radiology.179.3.2027985

[22] Levin TL , Liebling MS , Ruzal‐ Shapiro C , Berdon WE , Stolar CJ . Midgut malfixation in patients with congenital diaphragmatic hernia. What is the risk of midgut volvulus? Pediatr Radiol 1995; 25:259–261. 7567230 10.1007/BF02011092

[23] Luis AL , Hernandez F , Rivas S , et al. Midgut malrotation risk in abdominal wall defect. Cir Pediatr 2004; 17:125–128. 15503948

[24] Rescorla FJ , Shedd FJ , Grosfeld JL , Vane DW . Anomalies of intestinal rotation in childhood, analysis of 447 cases. Surgery 1990; 108:710–715. 2218883

[25] Applegate KE , Goske MJ , Pierce G , Murphy D . Situs revisited: imaging of the heterotaxy syndrome. Radiographics 1999; 19:837–852. 10464794 10.1148/radiographics.19.4.g99jl31837

[26] Cheikhelard A , De Lagausie P , Garel C , et al. Situs inversus and bowel malrotation: contribution of prenatal diagnosis and laparoscopy. J Pediatr Surg 2000; 35:1217–1219. 10945697 10.1053/jpsu.2000.8730

[27] Choi M , Borenstein SH , Hornberger L , Langer JC . Heterotaxia syndrome: the role of screening for intestinal rotation abnormalities. Arch Dis Child 2005; 90:813–815. 15890694 10.1136/adc.2004.067504 PMC1720530

[28] Ditchfield MR , Hutson JM . Intestinal rotational abnormalities in polysplenia and asplenia syndromes. Pediatr Radiol 1998; 28:303–306. 9569265 10.1007/s002470050358

[29] Wright JR , Barth RF , Neff JC , Poe ET , Sucheston ME , Stempel LE . Gastrointestinal malformations associated with Prune–Belly syndrome: three cases and a review of the literature. Pediatr Pathol 1986; 5:421–448. 3537997 10.3109/15513818609068868

[30] Filston HC , Kirks DR . Malrotation. The ubiquitous anomaly. J Pediatr Surg 1981; 16:614–620. 7277164 10.1016/0022-3468(81)90015-4

[31] Berdon WE , Baker DH , Blanc WA , Gay B , Santulli TV , Donovan C . Megacystis‐microcolon‐intestinal hypoperistalsis syndrome: a new cause of intestinal obstruction in the newborn. Report of radiologic findings in 5 newborn girls. Am J Roentgenol 1976; 126:957–964. 178239 10.2214/ajr.126.5.957

[32] Akkuş F , Doğru Ş . Superior mesenteric artery Doppler parameters in the evaluation of fetal hyperechogenic bowel. J Clin Ultrasound 2023; 51:1335–1341. 37589231 10.1002/jcu.23537

